# Peroxisome Deficiency in Cochlear Hair Cells Causes Hearing Loss by Deregulating BK Channels

**DOI:** 10.1002/advs.202300402

**Published:** 2023-05-12

**Authors:** Xiaolong Fu, Peifeng Wan, Ling Lu, Yingcui Wan, Ziyi Liu, Guodong Hong, Shengda Cao, Xiuli Bi, Jing Zhou, Ruifeng Qiao, Siwei Guo, Yu Xiao, Bingzheng Wang, Miao Chang, Wen Li, Peipei Li, Aizhen Zhang, Jin Sun, Renjie Chai, Jiangang Gao

**Affiliations:** ^1^ Medical Science and Technology Innovation Center Shandong First Medical University 250117 Jinan P. R. China; ^2^ State Key Laboratory of Digital Medical Engineering Department of Otolaryngology Head and Neck Surgery Zhongda Hospital School of Life Sciences and Technology Advanced Institute for Life and Health Jiangsu Province High‐Tech Key Laboratory for Bio‐Medical Research Southeast University 210096 Nanjing P. R. China; ^3^ School of Life Science Shandong University 266237 Qingdao P. R. China; ^4^ Department of Otolaryngology Head and Neck Surgery Affiliated Drum Tower Hospital of Nanjing University Medical School Jiangsu Provincial Key Medical Discipline (Laboratory) 210096 Nanjing P. R. China; ^5^ Department of Otorhinolaryngology Qilu Hospital of Shandong University NHC Key Laboratory of Otorhinolaryngology Shandong University 250012 Shandong Jinan P. R. China; ^6^ The First Affiliated Hospital of Suzhou University Suzhou University 215000 Suzhou P. R. China; ^7^ Co‐Innovation Center of Neuroregeneration Nantong University 226001 Nantong P. R. China; ^8^ Department of Otolaryngology Head and Neck Surgery Sichuan Provincial People's Hospital University of Electronic Science and Technology of China 610072 Chengdu P. R. China; ^9^ Institute for Stem Cell and Regeneration Chinese Academy of Science 101408 Beijing P. R. China; ^10^ Beijing Key Laboratory of Neural Regeneration and Repair Capital Medical University 100069 Beijing P. R. China

**Keywords:** BK channels, hair cells, peroxisome, Pex5

## Abstract

The peroxisome is a ubiquitous organelle in rodent cells and plays important roles in a variety of cell types and tissues. It is previously indicated that peroxisomes are associated with auditory function, and patients with peroxisome biogenesis disorders (PBDs) are found to have hearing dysfunction, but the specific role of peroxisomes in hearing remains unclear. In this study, two peroxisome‐deficient mouse models (*Atoh1‐Pex5^−/−^* and *Pax2‐Pex5^−/−^*) are established and it is found that peroxisomes mainly function in the hair cells of cochleae. Furthermore, peroxisome deficiency‐mediated negative effects on hearing do not involve mitochondrial dysfunction and oxidative damage. Although the mammalian target of rapamycin complex 1 (mTORC1) signaling is shown to function through peroxisomes, no changes are observed in the mTORC1 signaling in *Atoh1‐Pex5^−/‐^* mice when compared to wild‐type (WT) mice. However, the expression of large‐conductance, voltage‐, and Ca2^+^‐activated K^+^ (BK) channels is less in *Atoh1‐Pex5^−/−^* mice as compared to the WT mice, and the administration of activators of BK channels (NS‐1619 and NS‐11021) restores the auditory function in knockout mice. These results suggest that peroxisomes play an essential role in cochlear hair cells by regulating BK channels. Hence, BK channels appear as the probable target for treating peroxisome‐related hearing diseases such as PBDs.

## Graphical abstract

Hearing loss caused by peroxisome dysfunction in cochlear hair cells is not dependent on mitochondrial and oxidative stress pathways but on the down‐regulation of BK channel expression. Activation of BK channels rescues hearing in mice containing hair cells with dysfunctional peroxisomes, BK channels appear as the probable target for treating peroxisome‐related hearing diseases such as PBDs.

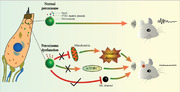

## 1 Introduction

Hearing loss is one of the most common sensory disorders in the world.^[^ ^1^ ^]^ According to the World Health Organization, one in five people in the world is hearing‐impaired.^[^ ^2^ ^]^ Cumulatively, hearing loss affects more than 1.5 billion people worldwide, and it is estimated that 2.5 billion people will experience some degree of hearing loss by 2050.^[^ ^2^ ^]^ In the absence of effective pharmaceutical compounds for prevention and treatment measures, hearing loss affects people's health and quality of life and causes societal economic losses.^[^ ^3^ ^]^ Sensorineural hearing impairment is the most common form of hearing impairment,^[^ ^4^ ^]^ and at present, effective therapeutic drug or gene therapy methods for the treatment of sensorineural hearing impairment are lacking. Therefore, a more mechanistic understanding of sensorineural hearing impairment is required to find relevant targets that can be used to prevent and treat the impairment.

Sensorineural hearing impairment is caused by a wide range of factors, including hair cell loss or dysfunction,^[^ ^5^ ^]^ spiral ganglion neurons (SGNs) degeneration,^[^ ^6^ ^]^ and stria vascularis (StV) atrophy.^[^ ^7^ ^]^ Nearly half of the cases of hereditary sensorineural hearing loss are associated with genetic mutations in hair cells.^[^ ^8^, ^9^ ^]^ Therefore, understanding the functional maintenance and survival of hair cells is the key to prevent sensorineural hearing impairment. In several previous studies, dysfunction of multiple hair cell organelles was associated with hearing loss. Lysosomal disorder was shown to result in increased basal autophagy, leading to progressive hair cell degeneration and hearing loss.^[^ ^10^, ^11^ ^]^ Endoplasmic reticulum (ER) stress could also lead to hair cell apoptosis and hearing loss,^[^ ^12^ ^]^ and the addition of salubrinal, an ER stress inhibitor, could delay hearing loss and preserve hair cells.^[^ ^13^ ^]^ Furthermore, mitochondrial dysfunction was also shown to increase oxidative stress and apoptosis of hair cells.^[^ ^14^, ^15^, ^16^, ^17^ ^]^ As an organelle commonly reported to have similar functions to mitochondria,^[^ ^18^ ^]^ the role of peroxisomes in hair cells has not been extensively studied. At present, only a few literature have reported that peroxisome may be involved in hearing protection. Pejvakin, encoded by *Pjvk* gene, plays a role in peroxisome proliferation, and *Pjvk^−/‐^* mice were found to be susceptible to noise.^[^ ^19^ ^]^ In our previous study, we also reported the involvement of peroxisomes in regulating the mammalian target of rapamycin complex 1 (mTORC1) signaling in the auditory system.^[^ ^20^ ^]^ A recent study showed that *Pex3*‐deficient mice (mice lacking *Pex‐3*, which codes for peroxisomal biogenesis factor 3) suffered from hearing loss.^[^ ^21^ ^]^ Furthermore, peroxisome biogenesis disorders (PBDs) are associated with hearing loss in clinical cases of peroxisome abnormalities.^[^ ^22^, ^23^ ^]^ However, a systematic investigation of peroxisomes’ role in cochlear hair cells is lacking.

**Figure 1:**
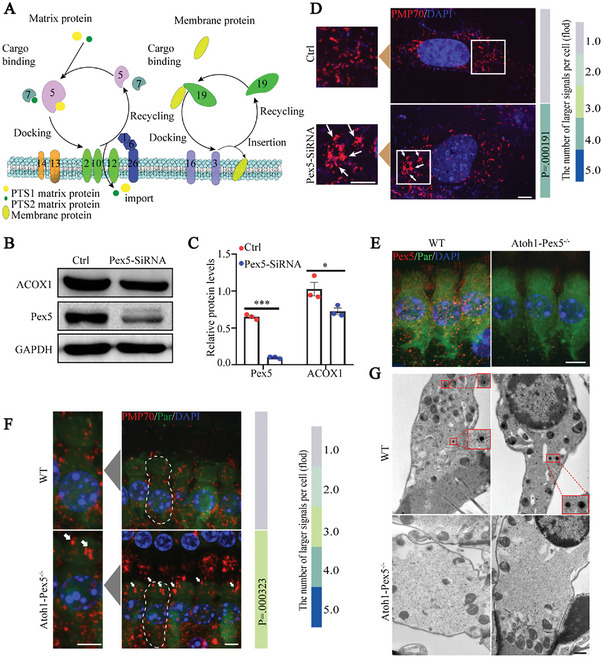
Pex5 knockout in mouse cochlear hair cells results in the absence of functional peroxisomes. A) Schematic representation of the process of peroxisomes biogenesis. The process of peroxisomes biogenesis is divided into two parts: One part is the transport process of matrix proteins. Among them, the PTS1 and PTS2 proteins are recognized by Pex5 and Pex7, respectively; Pex13‐Pex14 form the docking complex (brown); Pax2‐Pax10‐Pax12 form the ubiquitin ligase complex (light green); Pex1‐Pex6‐Pex26 form the export complex (dark blue). The other is those including Pex3, Pex16, and Pex19, responsible for the transport of peroxisome membrane proteins. Different numbers represent different proteins in the Pex family. B) Western blot of Pex5 and ACOX1 in HEI‐OC1 cells transfected with siRNA targeting Pex5 or control siRNA. C) Quantification of Pex5 and ACOX1 levels from (B); *n* = 3 for each group. D) Immunolabeling of PMP70 (red) and co‐staining with DAPI (blue) in HEI‐OC1 cells. Cells transfected with siRNA targeting Pex5 show a larger signal (arrows) than control cells. A local enlargement of the PMP70 signal is shown on the left. The number of these larger signals was quantified and shown on the right. Data are representative of three independent experiments. Scale bar 5 µm. (E) The mouse cochlea was immunolabeled with anti‐Pex5 (red), anti‐parvalbumin (green) and co‐stained with DAPI (blue). The Pex5 fluorescence was present in the hair cells of WT mice, but not in *Atoh1‐Pex5^−/‐^* mice, *n* = 4 for each group. Scale bar 5 µm. F) Immunolabeling of PMP70 (red), parvalbumin (green), and co‐staining with DAPI (blue) from 1‐month WT and *Atoh1‐Pex5^−/−^* mice. *Atoh1‐Pex5^−/−^* mice exhibited a larger signal in hair cells compared to WT mice. An individual hair cell is marked by a white dashed line and enlarged on the left. The signal was quantified and showed on the right, *n* = 90 cells from six mice. Scale bar 5 µm. G) Electron microscopy of hair cells from 1‐month WT and *Atoh1‐Pex5^−/−^* mice. The DAB‐labeled peroxisomes are indicated by a red box and enlarged. No DAB‐labeled peroxisomes were detected in the OHCs of *Atoh1‐Pex5^−/−^* mice in TEM analysis, *n* = 3 for each group. Scale bar 0.5 µm. Data represent the means ± SEM. * *p* < 0.05 and *** *p* < 0.001 by two‐tailed Student's t‐test.

Peroxisomes, which are round‐ or oval‐shaped membrane‐bound organelles, are found in almost all eukaryotic cells.^[^ ^24^ ^]^ Peroxisomes contain two main classes of proteins called a membrane and matrix proteins.^[^ ^25^ ^]^ These proteins are encoded by nuclear genes, synthesized on free cytosolic ribosomes, and translated to form peroxisomes ^[^ ^26^ ^]^ (**Figure** **1**A). Matrix proteins contain peroxisomal targeting signals (PTSs), which can be divided into PTS1 and PTS2.^[^ ^27^ ^]^ Most matrix proteins have PTS1 at the C‐terminus, whereas only a few matrix proteins contain PTS2.^[^ ^28^ ^]^ In the peroxisome biogenesis process, Pex5 plays an essential role in transporting PTS1‐containing matrix proteins.^[^ ^29^, ^30^, ^31^ ^]^ A deficiency of Pex5 leads to the loss of functional peroxisomes, resulting in membranous residuals called “ghosts”.^[^ ^32^ ^]^ Pex5 deficiency has also been associated with peroxisome‐related diseases.^[^ ^29^, ^33^, ^34^ ^]^ For example, Pex5 deficiency is known to cause demyelination and axonal degeneration in the central nervous system.^[^ ^35^, ^36^ ^]^ Furthermore, Pex5 deficiency causes hepatocyte hypertrophy and hyperplasia in the liver.^[^ ^37^ ^]^ Since Pex5 deficiency results in non‐functional peroxisomes,^[^ ^29^, ^36^, ^37^ ^]^ *Pex5*‐deficient mice represent an ideal model for studying the role of peroxisomes.^[^ ^38^ ^]^

This study, using *Atoh1‐Pex5^−/−^* and *Pax2‐Pex5^−/−^* mice, systematically examined the role of peroxisomes in cochlear hair cells. Peroxisomes were found to be essential for cochlear hair cells. Further, large‐conductance, voltage‐, and Ca2^+^‐activated K^+^ (BK) channels were identified as a possible target for the treatment of peroxisome‐associated hearing loss diseases such as PBDs.

## 2 Results

### 2.1 Pex5 Knockout in Mouse Cochlear Hair Cells Causes Peroxisomal Abnormalities

To investigate the role of peroxisomes in mouse cochlear hair cells, we first down‐regulated the Pex5 expression by transfecting siRNA to HEI‐OC1 cells (a mouse cochlear hair cell line), which led to an 84.7% decrease in the Pex5 levels in comparison to the control GSH, representing a good knockout efficiency (Figure 1B,C). In subsequent morphology and distribution analysis, control cells had a typical peroxisomal pattern, whereas Pex5 knockout cells showed an aggregation of peroxisome membrane protein 70 (PMP70) signals, which were larger than the peroxisome (Figure 1D). This was consistent with signals demonstrated in hepatocytes in a previous report.^[^ ^38^ ^]^ Studies have shown that the abnormal morphology and distribution of peroxisome reflect the dysfunction of peroxisomes.^[^ ^19^, ^38^ ^]^ To test the function of peroxisomes in Pex5 knockout cells, levels of acyl‐CoA oxidase 1 (ACOX1), whose reduced levels in its processed form indicate impaired transport capacity of matrix proteins,^[^ ^39^ ^]^ were examined. In comparison to the control cells, levels of the processed form of ACOX1 were significantly reduced (Figure 1B,C) in Pex5 knockout cells. Hence, Pex5 knockout in HEI‐OC1 cells caused peroxisomes to function abnormally.

To verify these results in vivo, *Pex5‐loxp* mice were crossed with mice expressing *Atoh1‐Cre*, generating mice with conditional *Pex5* knockout in the cochlear neurosensory epithelium (NSE). *Atoh1‐Cre* mouse line was reported to achieve efficient deletion of the *loxP*‐flanked regions in all inner ear hair cells and most of other supporting cells (SCs).^[^ ^40^ ^]^ Immunofluorescence and PCR analysis confirmed the *Pex5* knockout in hair cells (Figure 1E; Figure S1A, Supporting Information). *Atoh1‐Pex5^−/−^* mice showed immunofluorescence staining results identical to that obtained during in vitro analysis using HEI‐OC1 cells (Figure 1F). To explore the role of Pex5 in the sensory epithelium, we generated *Atoh1‐Cre/Pex5 ^fl/fl^/Rosa26‐tdTomato* mice, and tdTomato‐positive cells were isolated by flow cytometry. Similar to the results in cell lines, we detected significantly decreased levels of Pex5 and the processed form of ACOX1 (Figure S1B,C, Supporting Information), indicating impaired peroxisome function. Further, using 3,3‐diaminobenzidine (DAB) as the substrate, we conducted the catalase activity assay to label normally‐functioning peroxisomes in hair cells ^[^ ^19^, ^41^, ^42^ ^]^ and found that DAB labeled peroxisomes in hair cells of control mice, but not of *Atoh1‐Pex5^−/−^* mice (Figure 1G).

### 2.2 *Atoh1‐Pex5^‐/‐^* Mice Show Abnormal Hair Cell Function and Hearing Loss

**Figure 2:**
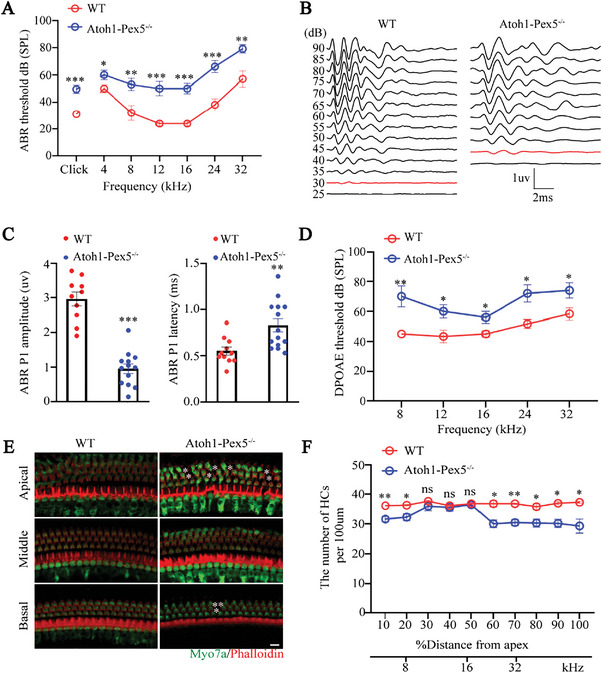
Abnormal hair cell function and hearing loss in *Atoh1‐Pex5^−/−^* mice. A) ABR threshold, B) ABR waveforms at click, C) ABR peak1 (P1) amplitude and latency, and D) DPOAE threshold of 1‐month WT and *Atoh1‐Pex5^−/−^* mice. *n* = 6−13 mice for each experiment. E) Immunofluorescence images of cochlea epithelial cells from 1‐month WT and *Atoh1‐Pex5^−/‐^* mice. Hair cells were labeled with Myo7a (green) and F‐actin were stained with Alexa Fluor 594‐Phalloidin (red), respectively. Losing hair cells are marked with a white asterisk. Scale bar 10 µm. F) Hair cell counts from 1‐month WT and *Atoh1‐Pex5^−/−^* mice, *n* = 3 for each group. Data represent the means ± SEM. “ns” represents not significant, * *p* < 0.05, ** *p* < 0.01, and *** *p* < 0.001, by two‐tailed Student's t‐test.

To explore whether peroxisome dysfunction in hair cells affects hearing in mice, we conducted an auditory brainstem responses (ABR) hearing test in 1‐month *Atoh1‐Pex5^−/−^* and control mice. *Atoh1‐Pex5^−/‐^* mice had significantly higher ABR thresholds at click and different frequencies (4, 8, 12, 16, 24, and 32 kHz) tested than that of control mice (**Figure** **2**A,B). When wave I of ABR was analyzed (Figure 2C), *Atoh1‐Pex5^−/‐^* mice had a lower wave I amplitude (32% of that observed in control mice) and larger wave I latency (0.83 ms in *Atoh1‐Pex5^−/−^* mice (*n* = 14) compared with 0.55 ms in control mice (*n* = 11)), implying that the function of inner hair cells (IHCs) was impaired in *Atoh1‐Pex5^−/‐^* mice. Moreover, the distortion product otoacoustic emission (DPOAE) results showed that the threshold of *Atoh1‐Pex5^−/‐^* mice was significantly increased than that of control mice (Figure 2D), suggesting that the function of outer hair cells (OHCs) was also impaired. When we used Myo7a and Phalloidin to label hair cells and stereocilia, respectively, 1‐month *Atoh1‐Pex5^−/−^* mice showed significant loss of OHCs in the apical and basal turns of the cochlea compared with littermate controls (Figure 2E,F). Interestingly, no significant difference was observed between the two groups in terms of the number of hair cells in the middle turn, with almost all hair cells still alive in *Atoh1‐Pex5^−/‐^* mice (Figure 2E,F). However, the ABR and DPOAE thresholds at the middle frequency (12 and 16 kHz) increased by 28.3 ± 1.7 dB compared to control mice, suggesting the abnormal function of hair cells. We further tested the number of hair cells in 3 and 5 months *Atoh1‐Pex5^−/‐^* mice and found degeneration of hair cells with increasing age of *Atoh1‐Pex5^−/‐^* mice than of wild‐type (WT) controls (Figure S2A,B, Supporting Information). These results suggest that mice with dysfunctional peroxisome in cochlear hair cells show abnormal hair cell function and hearing loss.

### 2.3 Peroxisomes is Not Essential in Other Cochlear Cell Types and Peroxisome Deficiency Does Not Affect Hair Cell Differentiation

**Figure 3:**
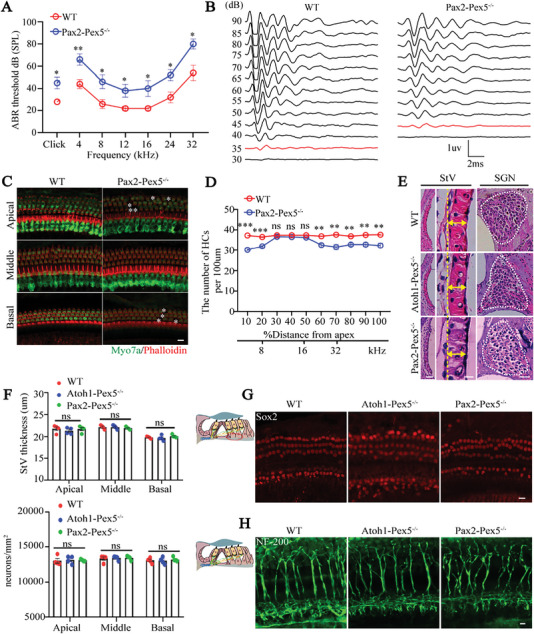
Peroxisomes function mainly in hair cells of the cochlea. A) ABR threshold in 1‐month WT and *Pax2‐Pex5^−/−^* mice, *n* = 5 for each group. B) Representative click ABR waveforms in 1‐month WT and *Pax2‐Pex5^−/−^* mice. ABR thresholds for each genotype are highlighted in red, *n* = 5 for each group. C) Confocal microscopy images of hair cells were labeled with Myo7a (green) and stained with Phalloidin (red), respectively. Losing hair cells are marked with a white asterisk. Scale bar 10 µm. D) Hair cell counts from 1‐month WT and *Atoh1‐Pex5^−/−^* mice, *n* = 4 for each group. E) Representative images of H&E staining of stria vascularis (StV) and spiral ganglion neurons (SGN) in basal turn of cochleae from 1‐month WT, *Atoh1‐Pex5^−/−^*, and *Pax2‐Pex5^−/−^* mice, *n* = 4 for each group. The figure on the left is an image of StV, the middle image shows a local enlargement of the StV (with bidirectional yellow arrows indicating the thickness of the StV), and the right image shows the SGN (with white dashed lines marking the area). Scale bar 40 µm, 10 µm, and 40 µm. F) Quantification of the thickness of the StV and the number of SGN from 1‐month WT, *Atoh1‐Pex5^−/−^*, and *Pax2‐Pex5^−/−^* mice, *n* = 4 for each group. G,H) Confocal images showed no differences in Sox2 for supporting cells and NF‐200 for neurofilament between WT, *Atoh1‐Pex5^−/−^*, and *Pax2‐Pex5^−/−^* mice, *n* = 3 for each group. The left is a model image of the organ of Corti, and the areas marked by red or green circle represent supporting cells or neurofilament, respectively. Scale bar 10 µm. Data represent the means ± SEM. “ns” represents not significant, * *p* < 0.05, ** *p* < 0.01, and *** *p* < 0.001, by two‐tailed Student's t‐test.

Atoh1‐mediated Cre expression started at embryonic day (E) 13.5; at this time, the hair cells have already differentiated. To explore the effects of peroxisome deficiency before hair cells differentiated, we crossed *Pax2‐Cre* (Cre expression started at E9.5) mice with *Pex5‐loxp* mice to generate *Pax2‐Pex5^−/−^* mice (Figure S3A, Supporting Information). *Pax2‐Pex5^−/−^* mice showed increased ABR and DPOAE thresholds, lower wave I amplitude and larger wave I latency, and slight loss of hair cells compared with WT mice, with no differences in comparison to *Atoh1‐Pex5^−/−^* mice (**Figure** **3**A–D; Figure S3B,C, Supporting Information). This result is interesting due to the fact that Pax2 and Atoh1 drive Cre expression just before and after the hair cell differentiation, respectively, but they exhibited similar hearing phenotypes. Furthermore, *Pax2‐Cre* drives Cre‐mediated recombination in most cochlear cell types containing peroxisomes, including SGNs, StV, hair cells, and SCs ^[^ ^43^ ^]^ (Figure S3D, Supporting Information). Consistently, the morphology of hematoxylin and eosin (H&E)‐stained cells, including SGNs and StV, was not significantly different in *Atoh1‐Pex5^−/‐^* and *Pax2‐Pex5^−/‐^* mice in comparison to WT mice (Figure 3E,F). In addition, the expression pattern of Sox2 (SCs’ marker) and NF‐200 (neurofilament marker) was not different among the three groups (Figure 3G,H). Furthermore, despite the strong presence of peroxisomes in the utricle system (Figure S3E, Supporting Information), the functional site of Atoh1 and Pax2, the development of stereocilia and hair cells in *Atoh1‐Pex5^−/−^* and *Pax2‐Pex5^−/−^* mice were comparable to that of WT mice (Figure S3F, Supporting Information), and *Atoh1‐Pex5^−/−^* and *Pax2‐Pex5^−/−^* mice did not show abnormal ambulatory behavior (data not shown). Collectively, these results indicate that peroxisomes mainly function in cochlear hair cells ofthe auditory system, and the peroxisome deficiency does not affect the differentiation of hair cells.

### 2.4 Hearing Loss Caused By Peroxisome Deficiency is Independent of Mitochondrial Defects and Oxidative Stress

**Figure 4:**
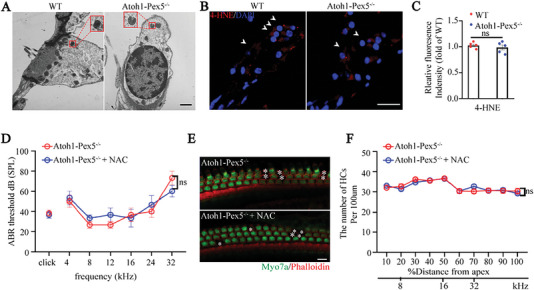
Hearing loss in *Atoh1‐Pex5^−/−^* mice is not due to mitochondrial and oxidative damage. A) TEM showed that 1‐month *Atoh1‐Pex5^−/−^* mice had normal mitochondrial structure in the hair cells, the mitochondria are also highlighted by a red box and enlarged. *n* = 3 for each group. Scale bar 1 µm. B) Immunolabeling of 4‐HNE (red) and co‐staining with DAPI (blue) in the organ of Corti of 1‐month WT and *Atoh1‐Pex5^−/−^* mice. The position of the hair cell is indicated by a white arrow. Scale bar 50 µm. C) Quantification of 4‐HNE fluorescence intensity from (B), *n* = 5 for each group. D) ABR thresholds in untreated and NAC‐treated 1‐month *Atoh1‐Pex5^−/−^* mice, *n* = 3 for each group. E) Immunofluorescence of cochlear epithelial cells from untreated and NAC‐treated 1‐month *Atoh1‐Pex5^−/−^* mice. Losing hair cells are marked with a white asterisk. Scale bar 10 µm. F) Hair cell counts in untreated and NAC‐treated *Atoh1‐Pex5^−/−^* mice, *n* = 3 for each group. Data represent the means ± SEM. “ns” represents not significant by two‐tailed Student's *t*‐test.

Peroxisomes are ubiquitous in rodent cells and share several functions with mitochondria, including in lipid metabolism ^[^ ^44^ ^]^ and activation of antiviral signals.^[^ ^45^ ^]^ Several studies have reported that peroxisome deficiency affects the mitochondrial integrity, resulting in abnormal mitochondrial structure and function.^[^ ^37^, ^46^, ^47^ ^]^ Notably, mitochondrial dysfunction is associated with increased oxidative stress and hearing loss in hair cells.^[^ ^14^, ^48^, ^49^ ^]^ Thus, we hypothesized that the hearing loss caused by peroxisome deficiency was due to a mitochondrial defect. Therefore, we examined mitochondrial morphology and reactive oxygen species (ROS) levels both *in‐vivo* and *ex‐vivo*. In immunofluorescence imaging using TOM20 (a mitochondrial membrane marker) antibody, we detected normal mitochondrial morphology and distribution in *Atoh1‐Pex5^−/−^* mice (Figure S4A, Supporting Information). Transmission electron microscopy (TEM) also revealed normal double‐membrane and prominent cristae structure of mitochondria in hair cells of *Atoh1‐Pex5^−/−^* mice (**Figure** **4**A). Furthermore, when we evaluated lipid peroxidation, using immunofluorescence‐based detection of 4‐hydroxynonenal (4‐HNE), which indicates ROS formation, no oxidative damage was detected in *Atoh1‐Pex5^−/−^* mice (Figure 4B,C). To further confirm that the hearing loss in *Atoh1‐Pex5^−/−^* mice was not due to oxidative stress, we administered *Atoh1‐Pex5^−/−^* mice with the antioxidant drug N‐acetyl cysteine (NAC) and found a significantly increased ratio of glutathione (GSH) to oxidized glutathione (GSSG). After NAC treatment, the expression of several antioxidant enzyme genes (such as *Gsr* and *Gpx2)* was significantly increased, while that of pro‐oxidant enzyme genes (such as *Lpo* and *Alox15*) was significantly decreased. These results suggested that NAC treatment enhanced the antioxidant defense ability of *Atoh1‐Pex5^−/‐^* mice in the cochleae (Figure S4C,D, Supporting Information). However, NAC treatment did not rescue the auditory phenotype of *Atoh1‐Pex5^−/−^* mice (Figure 4D), and no significant difference was observed in the number of hair cells between the NAC‐treated mice and control mice (Figure 4E,F). Consistently, no significant difference was observed in the mitochondria of *Pex5*‐knockout HEI‐OC1 cells and levels of Pex5 and 4‐HNE when compared to the control group (Figure S4E,F, Supporting Information).

### 2.5 Regulation of mTORC1 Signaling Does Not Affect Hearing of *Atoh1‐Pex5^−/−^* Mice

**Figure 5:**
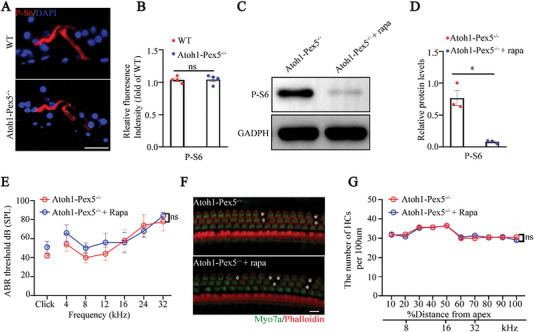
Hearing loss in *Atoh1‐Pex5^−/−^* mice was not related to mTORC1 signaling. A) Immunolabeling of P‐S6 (red) and staining with DAPI (blue) in the organ of Corti from 1‐month WT and *Atoh1‐Pex5^−/−^* mice. Scale bar 20 µm. B) Quantification of P‐S6 fluorescence intensity from (A), *n* = 4 for each group. C) Western blotting analysis of the cochlea of untreated and rapamycin‐treated *Atoh1‐Pex5^−/−^* mice. D) Quantification of P‐S6 protein levels from (C), *n* = 3 for each group. E) Comparison of ABR threshold between untreated and rapamycin‐treated *Atoh1‐Pex5^−/−^* mice, *n* = 5 for each group. F) Effects of rapamycin on hair cell survival in *Atoh1‐Pex5^−/−^* mice, losing hair cells are marked with a white asterisk. Scale bar 10 µm. G) Hair cell counts in untreated and rapamycin‐treated *Atoh1‐Pex5^−/−^* mice, *n* = 3 for each group. Data represent the means ± SEM. “ns” represents not significant, * *p* < 0.05, by two‐tailed Student's t‐test.

We previously showed that peroxisomes are involved in the regulation of mTORC1 signaling in the auditory system, and the overactivation of mTORC1 signaling led to premature death of cochlea hair cells.^[^ ^20^ ^]^ To test the effect of peroxisome dysfunction on the mTORC1 signaling, we examined the levels of S6 phosphorylation at 235/256 (P‐S6), a typical downstream target of mTORC1, and found no changes in P‐S6 levels in *Atoh1‐Pex5^−/−^* mice compared to the control mice, suggesting that the hearing loss in *Atoh1‐Pex5^−/−^* mice may not be related to mTORC1 signaling (**Figure** **5**A,B). Furthermore, when we treated *Atoh1‐Pex5^−/−^* mice with intraperitoneal rapamycin injection (1 mg kg^−1^; a widely used mTORC1‐specific inhibitor), an 89.8% reduction P‐S6 levels was observed in comparison to control mice, suggesting dramatically decreased mTORC1 levels in the cochleae of *Atoh1‐Pex5^−/−^* mice (Figure 5C,D). Previously, evidence showed that *Tsc1‐cKO* mice, a mouse model selectively activating mTORC1 signaling in NSE, exhibited hair cell loss and hearing loss, and their auditory function were effectively restored after treatment with rapamycin.^[^ ^20^ ^]^ Therefore, we hypothesized that if the hearing loss in *Atoh1‐Pex5^−/−^* mice is the result of mTORC1 over‐activation, treatment with rapamycin would have the same effect. However, the hearing level was not effectively improved in treated *Atoh1‐Pex5^−/−^* mice compared with untreated *Atoh1‐Pex5^−/−^* mice (Figure 5E), and no significant difference was observed in the number of hair cells between the two groups (Figure 5F,G).

### 2.6 Peroxisome Deficiency Leads to Abnormal BK Channels

**Figure 6:**
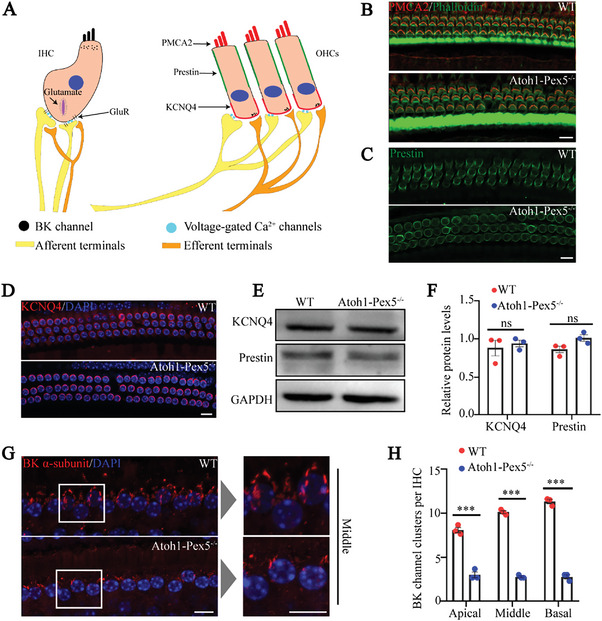
BK channel expression is decreased in cochlear hair cells of *Atoh1‐Pex5^−/−^* mice. A) Schematic representation of the distribution of several important proteins involved in hair cell function. There are three important proteins in the inner hair cells: Glutamate, GluR, and BK channels, and there are three important proteins in the outer hair cells: PMCA2, Prestin, and KCNQ4. Among them, voltage‐gated Ca^2+^ channels are distributed at the connection between the inner and outer hair cells at the afferent terminal. B) No significant difference was observed in the expression and distribution of PMCA2 between 1‐month WT and *Atoh1‐Pex5^−/−^* mice, *n* = 3 for each group. Scale bar 10 µm. C) No significant difference in the distribution and expression of Prestin protein in hair cells between 1‐month WT and *Atoh1‐Pex5^−/−^* mice, *n* = 5 for each group. Scale bar 10 µm. D) Immunofluorescence of KCNQ4 (red) from 1‐month WT and *Atoh1‐Pex5^−/−^* mice, *n* = 6 for each group. Scale bar 10 µm. E) Western blotting analysis of KCNQ4 and Prestin in the cochlea from 1‐month WT and *Atoh1‐Pex5^−/−^* mice. F) Quantification of KCNQ4 and Prestin levels from (E), *n* = 3 for each group. G) Immunofluorescence images of the BK *α*‐subunit of the cochlea sensory epithelium from 1‐month WT and *Atoh1‐Pex5^−/‐^* mice. The area selected by the white box is enlarged and displayed on the right side of the image. Scale bar 10 µm. H) BK‐channel expression in hair cells of 1‐month *Atoh1‐Pex5^−/‐^* mice was significantly reduced in apical, middle, and basal turns compared to 1‐month WT mice, *n* = 3 for each group. Data represent the means ±SEM. “ns” represents not significant, *** *p* < 0.001, by two‐tailed Student's *t*‐test.

Since peroxisome deficiency does not cause mitochondrial defects and oxidative stress, and has no relationship with mTORC1 signal, our next work was to explore how peroxisome dysfunction affects hair cell function. To this end, we analyzed the expression pattern of several classical proteins with hair cell function (**Figure** **6**A). PMCA2 is a plasma‐membrane calcium pump localized in the stereocilia or apical membrane of OHCs and functions as a transducer channel.^[^ ^50^ ^]^ Prestin is a motor protein located in the lateral membrane of OHCs and is essential for the amplification function of OHCs.^[^ ^51^ ^]^ KCNQ4 is a potassium channel located in the basal pole region of OHCs and plays an important role in potassium ion homeostasis.^[^ ^52^ ^]^ We did not detect any changes in the expression and distribution of these three proteins in *Atoh1‐Pex5^−/−^* mice (Figure 6, B‐D). Since the impaired function of IHCs or the number of ribbon synapses are the most common causes of ABR wave I abnormalities, we hypothesized that these two factors contribute to the hearing impairment in *Atoh1‐Pex5^−/−^* mice. Therefore, we evaluated otoferlin (an IHC exocytosis marker), ctbp2 (a presynaptic ribbon marker), and GluR2 (a postsynaptic marker), but found no changes in the expression and distribution of these proteins in IHCs of *Atoh1‐Pex5^−/−^* mice (Figure S5A–E, Supporting Information). Previous studies have reported that peroxisomes are essential for the synthesis of plasmalogen,^[^ ^53^ ^]^ and plasmalogen is involved in the regulation of BK channels in vascular smooth muscle cells.^[^ ^54^ ^]^ Notably, BK channels play a crucial role in the process of signal transduction in IHCs of the cochleae,^[^ ^55^ ^]^ and it has been shown that decreased expression of BK channels caused ABR wave I abnormalities and an increase in DPOAE threshold ;^[^ ^56^, ^57^, ^58^ ^]^ we interpreted that these phenotypes were similar to the auditory phenotypes of *Atoh1‐Pex5^−/−^* mice. Thus, we examined the expression of BK channels in the IHCs of *Atoh1‐Pex5^−/−^* mice and found that the *α*‐subunits of BK channels per IHC were lower in *Atoh1‐Pex5^−/−^* mice (2.85 ± 0.08, *n* = 270 IHCs from three mice) compared with control mice (9.84 ± 1.72, *n* = 270 IHCs from three mice) (Figure 6G,H). Collectively, these data demonstrate that peroxisomes deficiency in hair cells results in reduced the expression of BK channels and indicating dysregulation of BK channels might be the main reason for the hearing dysfunction of *Atoh1‐Pex5 ^−/−^* mice.

### 2.7 BK Channel Activation Rescues Hearing Dysfunction in *Atoh1‐Pex5^‐/‐^* Mice

**Figure 7:**
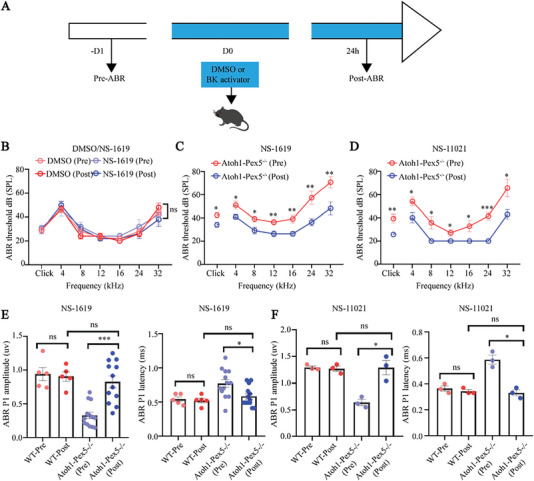
Activation of BK channels rescues hearing in *Atoh1‐Pex5^−/−^* mice. A) Timeline of BK activator treatment and ABR testing. NS‐1619 and NS‐11021 were administered intraperitoneally to 1‐month *Atoh1‐Pex5^−/−^* mice at doses of 4 mg kg^−1^ and 0.5 mg kg^−1^, respectively. B) ABR thresholds before and after treatment of DMSO or NS‐1619 in 1‐month WT mice, *n* = 5 for each group. C) ABR threshold before and after treatment with NS‐1619 (4 mg kg^−1^) in 1‐month *Atoh1‐Pex5^−/−^* mice, *n* = 11 for each group. D) ABR threshold before and after treatment with NS‐11021 (0.5 mg kg^−1^) in 1‐month *Atoh1‐Pex5^−/−^* mice, *n* = 7 for each group. E) ABR P1 amplitude and latency in WT and *Atoh1‐Pex5^−/‐^* mice before and after treatment with NS‐1619 (4 mg kg^−1^), *n* = 5−13 for each group. F) ABR P1 amplitude and latency in WT and *Atoh1‐Pex5^−/‐^* mice before and after treatment with NS‐11021 (0.5 mg kg^−1^), *n* = 3 for each group. Data represent the means ± SEM. “ns” represents not significant, * *p* < 0.05, ** *p* < 0.01, and *** *p* < 0.001, by two‐tailed Student's *t*‐test.

Since peroxisomes deficiency leads to dysregulation of BK channels, we wonder if dysregulation of BK channels is the main reason for the hearing disfunction of mutant mice. To confirm it, we intraperitoneally treated *Atoh1‐Pex5^−/‐^* mice with NS‐1619 (a common BK channel activator) ^[^ ^59^ ^]^ (**Figure** **7**A) at non‐toxic doses (4 mg kg^−1^) (Figure 7B) and found significantly decreased ABR thresholds after 24 h. In particular, the recovery of the hearing function at high frequencies (24 and 32 kHz) was the most significant; the ABR threshold decreased by 21.8 ± 0.9 dB after NS‐1619 treatment as compared to that before treatment (Figure 7C). This also implies that BK channels may contribute more toward high‐frequency hearing in mammals.^[^ ^60^ ^]^ Notably, the amplitude and latency of ABR wave I in *Atoh1‐Pex5^−/‐^* mice returned to normal level (*p* < 0.05, *n* = 5−13 for each group) (Figure 7E) after treatment. To further confirm that the hearing loss was indeed caused by the dysregulation of BK channels, we treated *Atoh1‐Pex5^−/−^* mice at non‐toxic concentration (0.5 mg kg^−1^; Figure S6A, Supporting Information) of another common BK channel activator NS‐11021 ^[^ ^61^ ^]^ and found effective rescue of hearing loss (Figure 7D,F). Further, NS‐11021 (ABR thresholds at click and 4−32 kHz after treatment were reduced by 15.3 dB and 17.2 ± 9.2 dB, respectively) showed better‐rescued potential in hearing in comparison to NS‐1619 (ABR thresholds at click and 4−32 kHz after treatment were reduced by 7.4 dB and 13.3 ± 5.2 dB, respectively), which could be attributed to better specificity and potency of NS‐11021.^[^ ^62^ ^]^ In addition to that, we found that restoration of auditory function in *Atoh1‐Pex5^−/‐^* mice with BK activators was not permanent, and the effective time was about 4 days (Figure S6B, Supporting Information). In conclusion, the hearing loss caused by peroxisome deficiency is due to BK channel dysregulation, which could be improved by BK channel activators.

## 3 Discussion

Although there are reports on the function of peroxisomes in other tissues and organs,^[^ ^35^, ^36^, ^37^ ^]^ recent evidence suggests an important role of peroxisomes in hearing. Moreover, patients with PBDs are also associated with hearing loss. However, the specific role of peroxisomes in hearing is still unclear.

To reveal the specific role of peroxisome in cochlear hair cells, we used two mouse models (*Atoh1‐Pex5^−/‐^* and *Pax2‐Pex5^−/−^*) and found that these mice developed hearing loss from 1‐month. We demonstrated that peroxisome is ubiquitously expressed in the cochlea and functions mainly in cochlear hair cells. Since we found that peroxisome deficiency causes hearing loss, we explored the mechanism behind this loss. Although mitochondrial defects, oxidative damage, and dependency on mTORC1 signaling were not related to hearing loss by peroxisome deficiency, dysregulation of BK channels was found to the main mechanism of hearing loss in such conditions.

The peroxisome is ubiquitous ^[^ ^24^ ^]^ and also widely distributed in different cell types of the cochlea. Interestingly, *Pax2‐Cre* expression is broader than *Atoh1‐Cre*. *Pax2‐Cre* also drives Cre‐mediated recombination in SCs, StV, and SGNs except for hair cells, but they share a similar hearing phenotype. This may be due to the following two reasons: ^[^ ^1^ ^]^ the energy metabolism in hair cells might be more active than that in other cell types.^[^ ^63^, ^64^ ^]^ Peroxisomes play an important role in cellular metabolism and contribute to the maintenance of intracellular energy homeostasis.^[^ ^65^ ^]^ In metabolically active cells, such as hepatocytes, it has been frequently reported that the loss of peroxisomes leads to the development of metabolic diseases.^[^ ^65^, ^66^ ^]^ However, the peroxisome deficiency does not affect the development and function of metabolically inactive cells, such as follicular B2 or T cells.^[^ ^32^ ^]^ In the present study, we found that peroxisomes mainly function in hair cells, which may be due to the unique role of peroxisomes in metabolically active cells with highly complex metabolic pathways that cannot be compensated by other organelles or signaling pathways. However, in other types of cells in cochlea, such as SCs, StV, and SGNs, the metabolic dysfunction caused by peroxisome deficiency may be compensated by other pathways, and ^[^ ^2^ ^]^ another possibility is that hair cells are more susceptible to changes in the external environment and are more vulnerable to damage. Studies have shown that hair cells are highly susceptible to changes in the cellular environment because they are the first cells to sense damage, such as noise exposure and aminoglycoside exposure.^[^ ^67^, ^68^ ^]^ Peroxisome deficiency leads to the imbalance of fatty acid oxidation, especially the accumulation of very‐long‐chain fatty acids,^[^ ^69^, ^70^ ^]^ and hair cells may be more prone to peroxisome deficiency‐mediated toxicity than other cell types.^[^ ^71^ ^]^ Overall, we found that peroxisomes only function in cochlear hair cells, and the reason of no effect of peroxisome deficiency in other cochlear cell types remains unclear and requires further investigation.

Peroxisomes share functional roles with mitochondria, including lipid metabolism and antiviral signal activation.^[^ ^44^, ^45^ ^]^ Several studies have reported that peroxisome deficiency disrupts the inner mitochondrial membrane and leads to oxidative stress.^[^ ^46^, ^47^ ^]^ However, in the present study, we did not detect defects in the mitochondrial structure, such findings have been made before when the presence of normal mitochondria was demonstrated in peroxisome‐deficient liver cells.^[^ ^37^ ^]^ This could be attributed to the heterogeneity of mitochondrial population in physiological and damaging conditions in different tissues. Moreover, mitochondria and peroxisomes are considered to be the main source of oxidative stress in inner ear cells,^[^ ^20^ ^]^ and mitochondrial damage can lead to oxidative damage,^[^ ^72^ ^]^ but we did not detect any oxidative damage in *Pex5* knockout HEI‐OC1 cells and cochlea hair cells of peroxisome‐deficient mice. Consistent with this finding, in a previous study, no oxidative stress was observed in patients with PBDs.^[^ ^73^ ^]^

In the present study, we found that peroxisome deficiency deregulates BK channels, a phenotype that can be rescued by treatment with BK channel activators. Hence, the observed hearing loss in *Atoh1‐Pex5^−/−^* mice was due to peroxisome deficiency‐mediated dysregulation of BK channels. Consistent with the results of the present study in *Atoh1‐Pex5^−/−^* mice, BK *α*‐subunit knockout mice were also reported to show identical hearing loss phenotype.^[^ ^57^ ^]^ Further, mutations in the BK‐encoding *sol* gene are a susceptibility factor for hearing loss in human.^[^ ^56^ ^]^ Of note, Pejvakin genes were found to be potentially associated with peroxisome proliferation, and abnormalities in BK channels were detected in *Pjvk^−/−^* mice.^[^ ^19^ ^]^ At this point, it is not clear whether the peroxisome deficiency affects BK channels directly or indirectly through another factor(s) or pathway(s). One of the important functions of peroxisomes is the synthesis of plasmalogens,^[^ ^74^ ^]^ and the activity of BK channels was associated with plasmalogens. Further, the treatment of mice with plasmalogens can slow neurodegeneration.^[^ ^75^ ^]^ More importantly, decreased levels of plasmalogens were observed in patients with PBDs.^[^ ^21^ ^]^ We speculate that peroxisomes might regulate the expression of BK channels through the synthesis of plasmalogens. Another possibility is that peroxisome deficiency leads to dysregulation of AMPK (AMP‐activated protein kinase) signaling,^[^ ^47^ ^]^ which is involved in the regulation of BK channels.^[^ ^76^ ^]^

**Figure 8:**
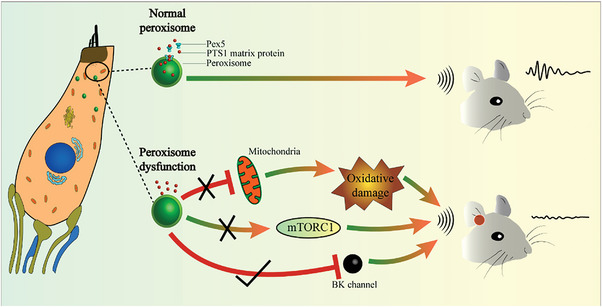
Model of peroxisome dysfunction in cochlear hair cells that leads to hearing loss. Hearing loss caused by peroxisome dysfunction in cochlear hair cells is not dependent on mitochondrial and oxidative stress pathways but on the down‐regulation of BK channel expression. Activation of BK channels rescues hearing in mice containing hair cells with dysfunctional peroxisomes.

In conclusion, this study for the first time systematically investigated the role of peroxisomes in cochlear hair cells using *Atoh1‐Pex5^−/−^* and *Pax2‐Pex5^−/−^* mice models. This study showed the important roles of peroxisomes in cochlear hair cells (**Figure** **8**), and we propose that the regulation of BK channels should be explored for the treatment of peroxisome‐associated hearing loss diseases such as PBDs.

## 4 Experimental Section

### Animals

*Pex5^fl/fl^* and *Rosa26‐tdTomato* mice were obtained from the Jackson laboratory. *Atoh1‐Cre* and *Pax2‐Cre* mice were gifts from Lin Gan (University of Rochester, New York, New York, USA).

The *Pex5^fl/fl^*, *Atoh1‐Cre*, and *Pax2‐Cre* mouse lines were backcrossed with the C57BL/6J line for six generations. *Atoh1‐Cre* and *Pax2‐Cre* mice were crossed with *Pex5^fl/fl^* mice. In the second generation, *Atoh1‐Pex5^fl/+^* and *Pax2‐Pex5^fl/+^* mice were bred with *Pex5^fl/fl^* mice. Further, *Atoh1‐Pex5^fl/fl^* and *Pax2‐Pex5^fl/fl^* mice were generated and named *Atoh1‐Pex5^−/−^* and *Pax2‐Pex5^−/−^* mice, mice that *Pex5^fl/+^ or Pex5^fl/fl^* without Cre were described as control mice in this study. The genotype of the offspring was identified by PCR of tail DNA using the following primer sequences: ^[^ ^1^ ^]^ *Atoh1‐Cre* (5’‐GCC TGC ATT ACC GGT CGA TGC‐3’ and 5’‐CAG GGT GTT ATA AGC AAT CCC‐3’), ^[^ ^2^ ^]^ *Pax2‐Cre* (5’‐GCC TGC ATT ACC GGT CGA TGC AAC GA‐3’ and 5’‐GTG GCA GAT GGC GCG GCA ACA CCA TT‐3’), and ^[^ ^3^ ^]^ *Pex5* (5’‐GTG GGG AAA GAA GGT GGA AG‐3’ and 5’‐CCT GCT TCG CTA CTG TTT GG‐3’). All animal experiments were approved by the Animal Ethics Committee of the School of Shandong First Medical University (No. 2023–126).

### Cell Culture and RNA Interference

Mouse cochlear hair cell line (HEI‐OC1) was cultured in DMEM (Dulbecco's modified eagle medium) supplemented with 10% fetal bovine serum at 33 °C under 10% CO_2_. siRNA (GenePharma) was used to knock down Pex5. Cells were transfected with siRNA oligonucleotides using the GP‐transfect‐Mate (GenePharma). Cells were incubated for 72 h before use. The siRNA sequences were as follows: 5’‐CCC UGU UUC UUG AAG UAA ATT‐3’ and 5’‐ UUU ACU UCA AGA AAC AGG GTT‐3’. The negative control sequences were 5’‐UUC UCC GAA CGU GUC ACG UTT‐3’ and 5’‐ACG UGA ACA GUU CGG AGA ATT‐3’.

### ABR and DPOAE Measurements

The ABR and DPOAE thresholds were measured as described previously.^[^ ^20^ ^]^ Briefly, mice were intraperitoneally injected with pentobarbital sodium (50 mg kg^−1^), and the temperature of the mice was maintained at 37 °C using a heating pad for the further measurement of ABR and DPOAE. For ABR recordings, three needle electrodes were inserted into the top of the skull between the ears of mice below the right auricle and dorsal midline. ABR thresholds were recorded at different stimulus frequencies (4, 8, 12, 16, 24, and 32 kHz) using a Tucker‐Davis Technologies (TDT) System and BioSigRZ software. The intensity of sound varied from 90 dB to 10 dB, decreasing every 5 dB. The lowest sound intensity at which an auditory response could be detected was defined as the mouse hearing threshold. The DPOAE of 2f1‐f2 was measured using the Real‐Time Signal Processing System II from TDT, with f2/f1 = 1.22 and the f2 5 dB lower than f1. The DPOAE threshold was defined as a peak at 2f2‐f1 when 3 dB above the noise floor. The amplitude and latency of ABR wave I were analyzed using MS excel and BioSigRZ softwares. Hearing assessment was performed in each group of mice using a blinded procedure.

### Quantitative Real‐Time PCR (qPCR)

According to the manufacturer's instructions, total RNA was extracted from the cochlea of one‐month‐old *Atoh1‐Pex5^−/−^* mice treated with or without NAC using Trizol reagent (Invitrogen, 15 596 026). Then, cDNA was synthesized using a reverse transcription kit (Vazyme, R323). Subsequently, qPCR was performed with a mixture containing 10ul SYBR Green qPCR Mix (Vazyme, Q711), 0.4ul Primer F, 0.4ul Primer R, 1ul Template cDNA, and 8.2ul of ddH_2_O. Each sample was run in triplicate, and the relative expression was calculated using the 2^−ΔΔCT^ method. The primer sequences used are as follows: GAPDH (F: 5’‐GACTTCAACAGCAACTC‐3’; R: 5’‐ CTTGCTCAGTGTCCTTGCTG‐3’), Gsr (F: 5’‐ TATGTGAGCCGCCTGAACA‐3’; R: 5’‐ GTGGCAATCAGGATGTGTGG‐3’), Gpx2 (F: 5’‐ GAACAACTACCCGGGACTAC‐3’; R: 5’‐ GTCGGACATACTTGAGGCTG‐3’), Lpo (F: 5’‐ CTGGACCAGAAGAGATCCATG‐3’; R: 5’‐ TCACCAGGTGGGAACATGATGG‐3’), Alox15 (F: 5’‐ GACTTGGCTGAGCGAGGACT‐3’; R‐5’‐ CTTGACACCAGCTCTGCA‐3’).

### Determination of Glutathione (GSH) and Oxidized Glutathione (GSSG) Contents

According to the manufacturer's instructions, using a glutathione assay kit to measure the levels of GSH and GSSG.^[^ ^19^ ^]^ The total glutathione and GSSG levels are measured by spectrophotometry at 405 nm. The GSH levels are calculated by subtracting twice the GSSG concentration from the total glutathione concentration.

### Western Blot

For cell lysis, medium‐strength RIPA lysis buffer (89 900, Thermo Fisher scientific), protease inhibitors mixture (P0100, Solarbio), and protein phosphatase inhibitor (P1260, Solarbio) were added to the cells, and the cells were placed on ice for 30 min. The samples were subsequently centrifuged at 14 000 × *g* for 10 min at 4 °C, and the supernatant was collected, supplied with SDS denaturing buffer, boiled for 10 min, and subjected to SDS‐PAGE and western blotting analysis after transferring onto PVDF (Polyvinylidene fluoride) membranes (IPVH00010, Millipore). The membranes were blocked with 5% skim milk (P0216, Beyotime) in TBST for 2 h and then incubated overnight at 4 °C with primary antibodies diluted in 5% BSA (A8020, Solarbio) prepared in TBST. Subsequently, membranes were washed three times (10 min each) with TBST, followed by incubation with secondary antibodies (diluted in 5% skim milk) for 1 h at room temperature. ECL (E412‐01, Vazyme) substrate was added to visualize the signal. For protein extraction from the cochlear tissues of sacrificed mice (using excessive anesthesia), the temporal bones were quickly removed, placed in a mixture of medium strength RIPA lysates buffer, protease inhibitor, and protein phosphatase inhibitor, thoroughly grounded and lysed on ice for 30 min, and centrifuged at 14 000 × *g* for 10 min at 4 °C. The supernatant was collected and subjected to SDS‐PAGE and western blotting analysis as described above. The following primary antibodies were used: ACOX1 (ab184032, Abcam, 1:1000), Pex5 (NBP1‐87185, Novus Biologicals, 1:1000), P‐S6 (Ser 235/236) (4858T, CST, 1:2000), KCNQ4 (SMC‐309, StressMarq Biosciences, 1:1000), Prestin (sc‐22694, Santa Cruz, 1:1000), 4‐HNE (ab46545, Abcam, 1:1000), Ctbp2 (612 044, BD Biosciences, 1:1000), and GADPH (AF7021, Affinity, 1:10 000).

### Immunofluorescence analysis

After rapidly dissecting the temporal bones in cold PBS, the cochleae were fixed overnight in 4% paraformaldehyde at 4 °C and decalcified the next day in 10% EDTA at room temperature for 1 day. For paraffin sections, decalcified cochleae were dehydrated using ethanol and immersed in paraffin. After sectioning, the sections were deparaffinized and hydrated, immersed in unmasking solution, and boiled for 10 min. Sections were subsequently incubated in blocking solution (10% goat or donkey serum in PBS) for 30 min at room temperature, followed by overnight incubation at 4 °C in primary antibodies diluted in PBS. The next day, the sections were washed three times with PBS for 5 min each. Sections were subsequently incubated with secondary antibodies diluted in PBS for 1−2 h at room temperature. For immunofluorescence analysis of the basement membrane, the decalcified cochlea was dissected using a microscope to isolate the basement membrane. Subsequently, the membrane was permeabilized using 0.25% Triton‐X‐100 for 15 min at room temperature, and then immersed in a blocking solution and incubated for 30 min at room temperature. The basement membrane was incubated overnight in PBS containing the primary antibody at 4 °C, washed three times with PBS the next day, and incubated in the secondary antibody for 1−2 h at room temperature. Cochlear samples were visualized using a confocal microscope (ZEISS LSM 900). The following antibodies were used: PMP70 (ab3421, Abcam, 1:200), Parvalbumin (MAB1572, Sigma, 1:2000), Pex5 (NBP1‐87185, Novus Biologicals, 1:200), Myo7a (25‐6790, Proteus Biosciences, 1:200), Sox2 (ab97959, Abcam, 1:200), NF‐200 (ab4680, Abcam, 1:200), 4‐HNE (1:100, Abcam, ab46545), P‐S6 (Ser 235/236) (4858T, CST, 1:200), PMCA2 (PA1‐915, ThermoFisher Scientific, 1:200), Prestin (sc‐2294, Santa Cruz, 1:200), KCNQ4 (SMC‐309, StressMarq Biosciences, 1:200), KCNMA1 (APC‐107, Alomone Labs, 1:200), TOMM20 (ab56783, Abcam, 1:200), otoferlin (ab53233, Abcam, 1:200), Ctbp2 (612 044, BD Transduction Laboratories, 1:200), GluR2 (MAB397, Millipore, 1:200), and Phalloidin‐iFluor 594 (ab176757, Abcam, 1:2000).

### Hematoxylin and Eosin Staining

The slides were immersed in xylene solution for 30 min, followed by gradient rehydration in ethanol, and placed in distilled water for 3 min. They were then incubated with hematoxylin for 3 min, washed with distilled water for 3 min, incubated with 1% hydrochloric acid alcohol for 5 s, washed with distilled water for 3 min, again incubated with eosin, dehydrated in graded ethanol (80%, 95%, and 100%), immersed in xylene solution, coverslipped, and eventually visualized using the light microscope (OLYMPUS BX53).

### Transmission Electron Microscopy

For observation of mitochondrial morphology, cochlea of WT and *Atoh1‐Pex5^−/−^* mice were fixed in 1% glutaraldehyde at 4 °C. The basal membrane of cochlea was dissected under a microscope after decalcification in 10% EDTA, fixed for 2 h in 1% tetroxide at 4 °C, washed in deionized water, dehydrated in graded ethanol, embedded in Epon 812 resin, and visualized under the electron microscope. The DAB method was used for selective staining of peroxisomes.^[^ ^19^, ^41^, ^42^ ^]^ Here, the cochlea was fixed in 1% glutaraldehyde (at pH 7.2 and 4 °C) for 1 h, washed 3 times with 0.1 m cacodylate buffer, and incubated in 10 mM 3,3’‐diaminobenzidine (DAB) (D5637, Sigma) and 0.15% H_2_O_2_ in 0.05 m Teorell−Stenhagen buffer (pH 10.5 and 30 °C) for 45 min. The sample was washed three times with 0.05 m Teorell−Stenhagen buffer and then fixed in 1% tetroxide for 2 h at 4 °C. This was followed by washing in deionized water, dehydration in graded ethanol, embedding with Epon 812 resin, and eventual visualization under the electron microscope.

### NAC Treatment

As previously reported,^[^ ^19^ ^]^ *Atoh1‐Pex5^−/−^* pregnant mice were treated (through drinking water) with 1% NAC. *Atoh1‐Pex5^−/−^* pups were treated with 1 m NAC in uteri and breast milk. The hearing of the pups was measured at 1 m. Each cage contained three pups.

### Rapamycin treatment

Rapamycin (20 mg mL^−1^) was dissolved in methanol and stored at ‐20 °C. The working solution (0.1 mg kg^−1^) was prepared after diluting in 0.25% Tween‐80 and 0.25% PEG‐300. Mice were intraperitoneally injected (1 mg kg^−1^) every other day from 14^th^ day until 1 month. The hearing function and hair cell count were determined after 1 month.

### Treatment with activators of BK channels

NS‐1619 and NS‐11021 are the two most commonly used activators of BK channels. These were used at non‐toxic doses determined previously.^[^ ^62^, ^77^ ^]^ According to the manufacturer's instructions, NS‐1619 and NS‐11021 should first be dissolved in DMSO to prepare a stock solution of 1 mg mL^−1^ and stored at ‐80 °C. The working concentration (0.1 mg mL^−1^) is obtained by diluting with physiological saline, and should be freshly prepared before each use and used on the same day. Mice (at 1 month) were intraperitoneally injected with 4 mg kg^−1^ and 0.5 mg kg^−1^ of NS‐1619 and NS‐11021, respectively. *Atoh1‐Pex5^−/−^* mice were divided into DMSO, NS‐1619, and NS‐11021 groups, and the ABR of each group was measured before and 24 h after administration.

### Statistical analysis

All data are expressed as the means ± standard error of the mean of at least three independent experiments, and two‐tailed Student's *t*‐test was used to determine the significance between groups. All data were analyzed using GraphPad Prism 8.0 software. *p* < 0.05 was considered statistically significant.

## Conflict of Interest

The authors declare no conflict of interest.

## Author Contributions

X.F., R.C., and J.G. designed the project. P.W., L.L., Y.W., Z.L., G.H., S.C., X.B., J.Z., R.Q., S.G., Y.X., B.W., M.C., W.L., P.L., A.Z., and J.S. performed the experiments and acquired the data. P.W., X.F., and Y.W. analyzed the results and wrote the manuscript.

## Supporting information

Supporting Information

## Data Availability

The data that support the findings of this study are available in the supplementary material of this article.
