# Supplementary material for: Peroxisome Deficiency in Cochlear Hair Cells Causes Hearing Loss by Deregulating BK Channels
Source: Adv Sci (Weinh). 2023 May 12;10(20):2300402. doi: 10.1002/advs.202300402 (PMC10369297; doi:10.1002/advs.202300402)
Supplement: Supplementary file 1 — Supporting Information [file ADVS-10-2300402-s001.pdf]

ADVANCED SCIENCE
Open Access

Supporting Information

for *Adv. Sci.*, DOI 10.1002/advs.202300402

Peroxisome Deficiency in Cochlear Hair Cells Causes Hearing Loss by Deregulating BK Channels

Xiaolong Fu, Peifeng Wan, Ling Lu, Yingcui Wan, Ziyi Liu, Guodong Hong, Shengda Cao, Xiuli Bi, Jing Zhou, Ruifeng Qiao, Siwei Guo, Yu Xiao, Bingzheng Wang, Miao Chang, Wen Li, Peipei Li, Aizhen Zhang, Jin Sun, Renjie Chai* and Jiangang Gao**

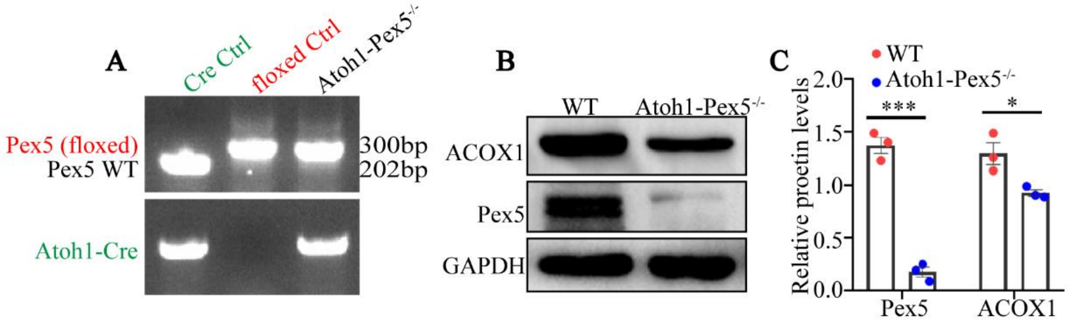

Supplemental Figure 1 Peroxisome dysfunction in cochlear hair cells of *Atoh1-Pex5*$^{-/-}$ mice. **(A)** Genotypes of control and *Atoh1-Pex5*$^{-/-}$ mice. **(B)** Western blotting analysis of ACOX1 and Pex5 in tdTomato-positive cells isolated by flow cytometry. **(C)** Quantification of Pex5 and ACOX1 protein levels from (B), n=3 for each group. Data represent the means ± SEM. * P<0.05 and *** P<0.001, by two-tailed Student's t-test.

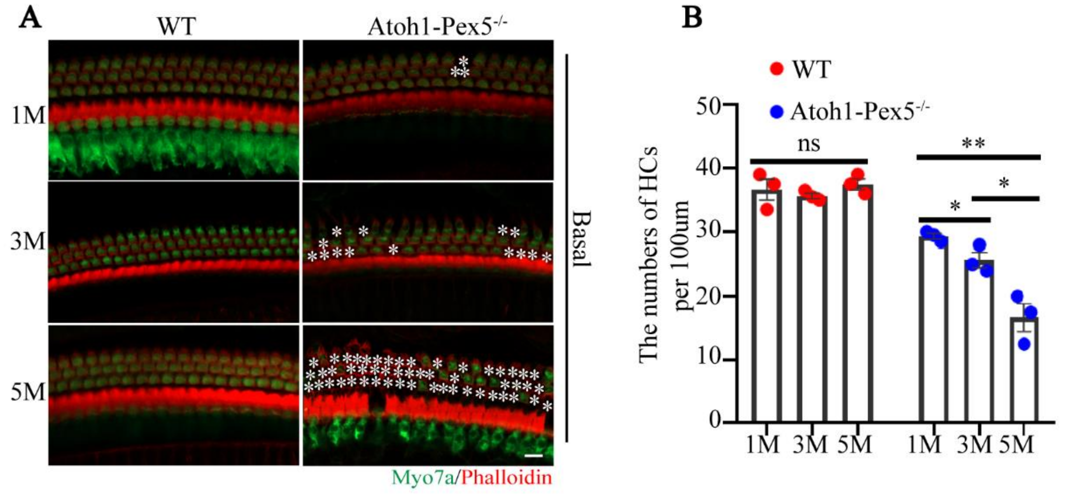

Supplemental Figure 2 *Atoh1-Pex5*$^{-/-}$ mice show increased hair cell loss with age. (A) Results of basement membrane from 1-, 3-, and 5-month WT and *Atoh1-Pex5*$^{-/-}$ mice. Hair cells were labeled with Myo7a (green) and F-actin were stained with Phalloidin (red), respectively. Losing hair cells are marked with a white astreisk. Scale bar 10 µm. **(B)** Hair cell counts in the basal turn of 1-, 3-, and 5-month WT and *Atoh1-Pex5*$^{-/-}$ mice. Data represent the means ± SEM. "ns" represents not significant, * P<0.05 and ** P<0.01, by two-tailed Student's t-test.

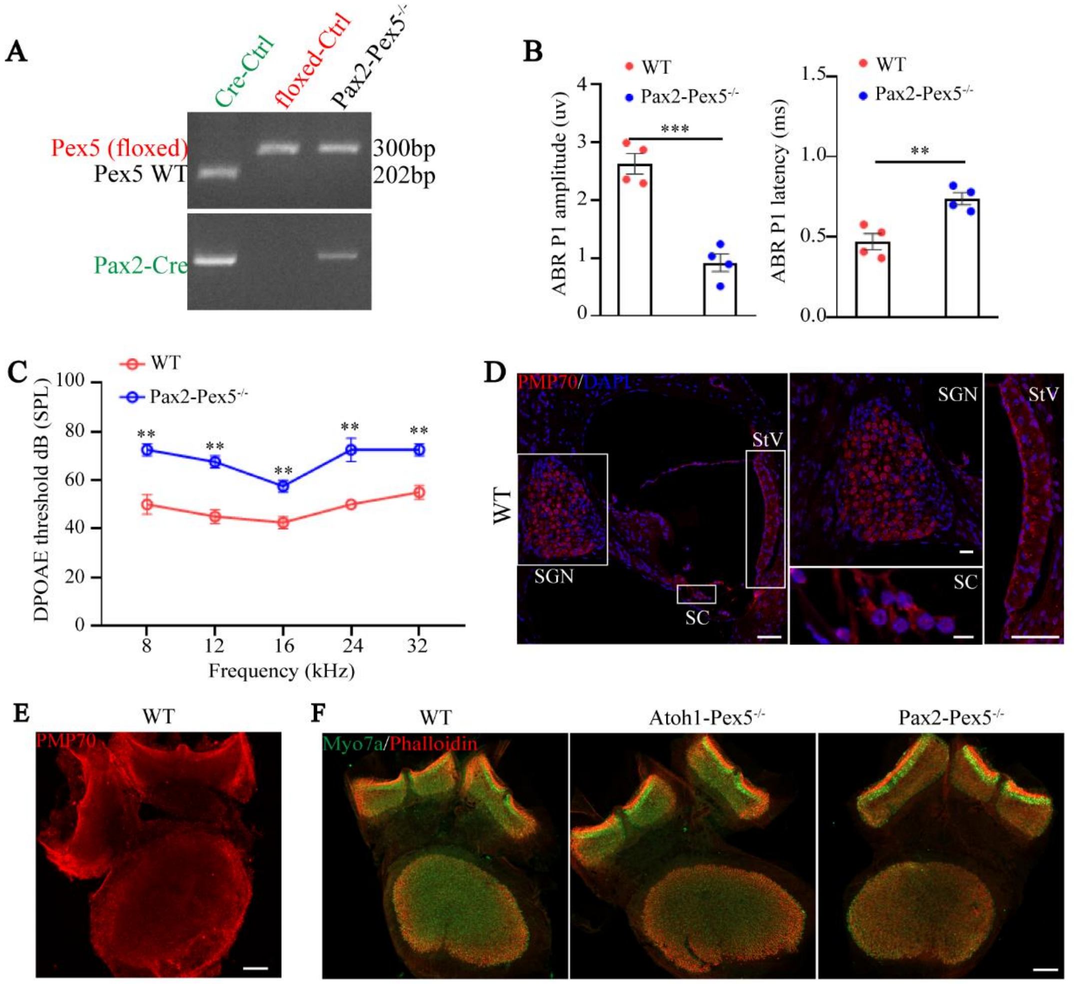

Supplemental Figure 3 The SGN, StV, and vestibule of *Pax2-Pex5$^{-/-}$* mice are not affected. (A) Genotypes of control and *Pax2-Pex5$^{-/-}$* mice. **(B)** ABR peak1 (P1) amplitude and latency, and **(C)** DPOAE threshold of 1-month WT and *Pax2-Pex5$^{-/-}$* mice. Compared to WT mice, *Pax2-Pex5$^{-/-}$* mice showed a lower wave I amplitude (35% of control mice), a larger wave I latency (0.74 ms in *Pax2-Pex5$^{-/-}$* mice compared to 0.47 ms in WT mice), and a higher DPOAE threshold. n=4 mice for each experiment. **(D)** Immunofluorescence images of cochlear section from 1-month WT mice showed peroxisomes distributed in SC, SGN, and StV. The SC, SGN and StV regions indicated by the white box and enlarged on the right. Scale bar 50 µm, 20 µm, and 20 µm. SC: Supporting cell; SGN: spiral ganglion neurons; StV: stria vascularis. **(E)** Immunofluorescence images of 1-month WT mice showed that peroxisomes were distributed in the vestibule. Scale bar 100 µm. **(F)** No significant difference was observed in the vestibule between WT, *Atoh1-Pex5$^{-/-}$*, and *Pax2-Pex5$^{-/-}$* mice, n=4 for each group. Scale bar 100 µm. Data represent the means ± SEM. ** P<0.01, *** P<0.001, by two-tailed Student's t-test.

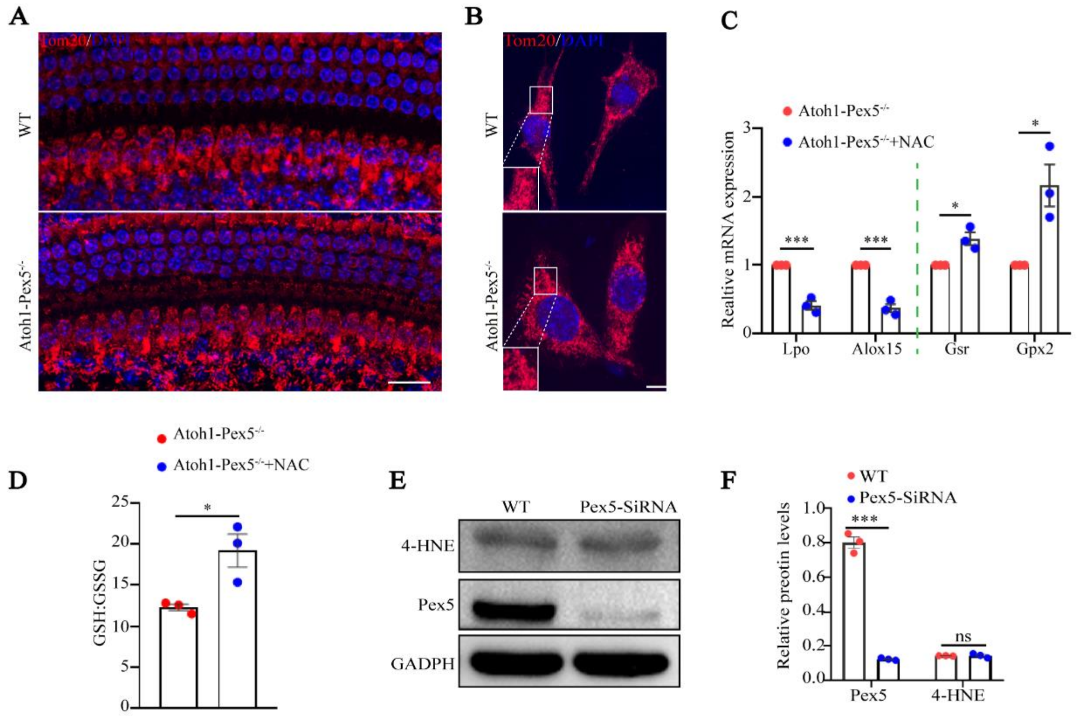

Supplemental Figure 4 Hearing loss in *Atoh1-Pex5*$^{-/-}$ mice is not related to mitochondrial and oxidative stress. (A) No significant difference was observed in the mitochondria of hair cells from 1-month WT and *Atoh1-Pex5*$^{-/-}$ mice, n=4 for each group. Scale bar 10 μm. **(B)** Immunofluorescence images of TOM20 (red) and DAPI (blue) in HEI-OC1 cells. The region indicated by the white box is further enlarged. Data are representative of four independent experiments. Scale bar: 10 μm. **(C)** Quantitative real-time PCR results showed that the expression of some antioxidant enzyme genes (*Gsr* and *Gpx2*) was significantly increased after NAC treatment, and the genes for pro-oxidant enzymes (*Lpo* and *Alox15*) were significantly decreased in NAC-treated *Atoh1-Pex5*$^{-/-}$ mice compared to NAC-untreated group, n=3 for each group. **(D)** The ratio of glutathione (GSH) to oxidized glutathione (GSSG) in NAC-treated *Atoh1-Pex5*$^{-/-}$ mice was significantly higher than that in NAC-untreated *Atoh1-Pex5*$^{-/-}$ mice, n=3 for each group. **(E)** Western blotting analysis of Pex5 and 4-HNE in HEI-OC1 cells transfected with siRNA targeting Pex5 or control siRNA. **(F)** Quantification of Pex5 and 4-HNE levels from (E), n=3 for each group. Data represent the means ± SEM. "ns" represents not significant, * $P<0.05$, *** $P<0.001$, by two-tailed Student's t-test.

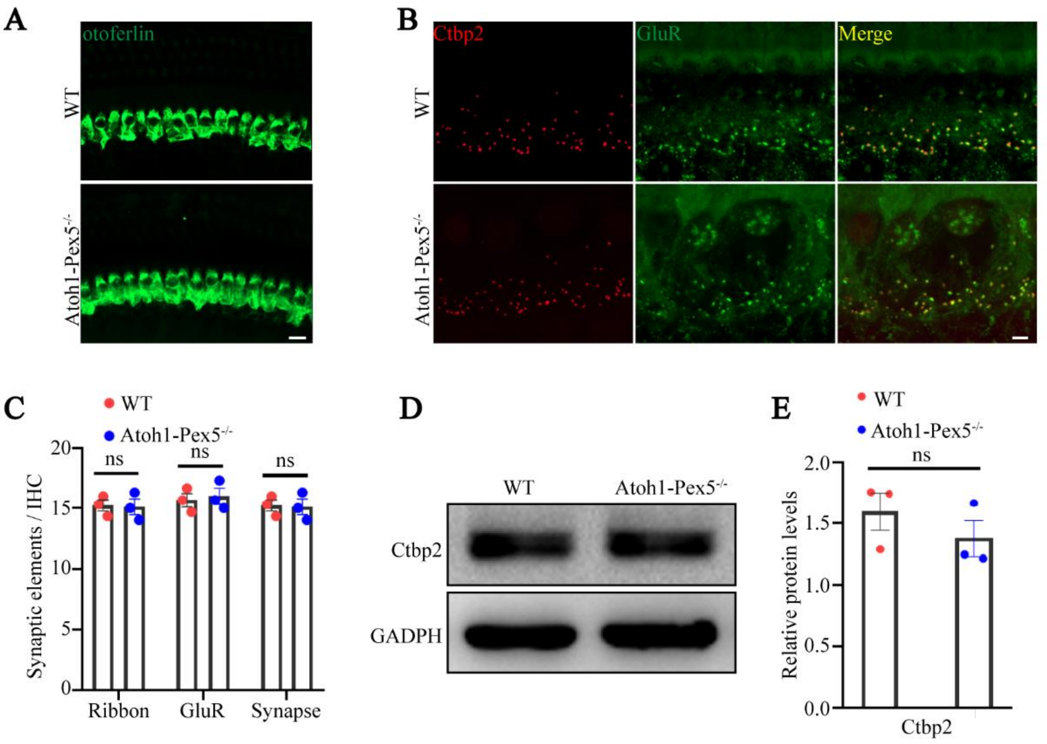

Supplemental Figure 5 The otoferlin expression pattern and the number of ribbon synapses are not affected in *Atoh1-Pex5$^{-/-}$* mice. (A) Immunofluorescence images of otoferlin in 1-month WT and *Atoh1-Pex5$^{-/-}$* mice. **(B)** Immunofluorescence images of ribbon synapses from 1-month WT and *Atoh1-Pex5$^{-/-}$* mice. The presynaptic ribbon and postsynaptic GluR were labeled with anti-ctbp2 (red) and anti-GluR (green) antibodies, respectively. Scale bar 5 µm. **(C)** Quantification analysis of ribbon, GluR, and synapse, n=3 for each group. **(D)** Western blotting analysis of cochleae from 1-month WT and *Atoh1-Pex5$^{-/-}$* mice. **(E)** Quantification of ctbp2 levels from (D), n=3 for each group. Data represent the means ± SEM. “ns” represents not significant, by two-tailed Student’s t-test.

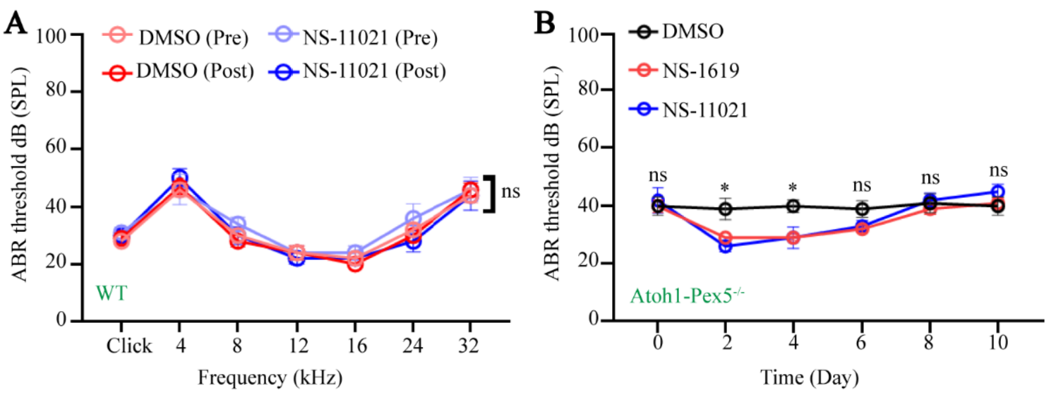

Supplemental Figure 6 Determination of BK activator drug duration in *Atoh1-Pex5*$^{-/-}$ mice. **(A)** ABR thresholds before and after treatment of DMSO or NS-11021, n=5 for each group. **(B)** Detection of ABR hearing thresholds (Day 0, 2, 4, 6, 8, 10) in *Atoh1-Pex5*$^{-/-}$ mice treated with two BK activators, NS-1619 and NS-11021, n=5 for each group. Data represent the means ± SEM. "ns" represents not significant, * P<0.05, by two-tailed Student's t-test.
